# Short-term acclimation at elevated temperatures alters hematobiochemical parameters, erythrocyte and muscle structure, and critical thermal tolerance (CTmax) limit of Nile tilapia, *Oreochromis niloticus*

**DOI:** 10.1007/s00360-025-01650-z

**Published:** 2026-02-06

**Authors:** S.M. Majharul Islam, Md. Shahjahan, Umme Khadiza Noon, Ioannis N. Vatsos

**Affiliations:** 1 Faculty of Biosciences and Aquaculture, Nord University, 8049 Bodø, Norway; 2 Laboratory of Fish Ecophysiology, Department of Fisheries Management, Bangladesh Agricultural University, Mymensingh, 2202 Bangladesh

**Keywords:** Nile tilapia, Temperature, Thermal tolerance, Muscle architecture, Hematology

## Abstract

Rising water temperatures pose a significant challenge for the aquaculture industry. These increased temperatures negatively affect fish survival and restrict various physiological processes. This study aimed to assess multiple physiological responses and identify the critical thermal tolerance limits (CTmax) of Nile tilapia subjected to acute thermal stress at different acclimation temperature regimes. In this study, fish were acclimated to three temperature regimes —31 °C, 34 °C, and 37 °C — for 1 week. To determine the CTmax, muscle microstructure was evaluated at the end, and several major hemato-biochemical parameters were measured at the beginning and end of the acute thermal stress, just before the fish lost equilibrium. The results indicated a significant decrease in Hb and RBC levels, while WBC and glucose levels increased notably at 37 °C. The frequencies of erythrocyte cellular abnormalities (ECAs) and erythrocyte nuclear abnormalities (ENAs) were also considerably higher at 37 °C than at the other temperature groups. Muscle fibres were mostly elongated and irregularly shaped at 37 °C compared with the other temperature groups. The CTmax was significantly lower at 37 °C acclimation (41.15 °C) than at 31 °C (44.57 °C) and 34 °C (43.40 °C). Principal component analysis revealed that acute thermal stress at the acclimated temperature (37 °C) significantly affected the hematobiochemical parameters at the endpoint. Furthermore, a negative correlation between CTmax and temperature was observed, indicating that CTmax decreased significantly (p < 0.05) with increasing temperature. From this study, it can be concluded that *Oreochromis niloticus* should be maintained at temperatures below 37 °C to ensure optimal health conditions.

## Introduction

Temperature plays a crucial role in influencing the development and physiology of fish, thereby impacting aquaculture production. (Ashaf-Ud-Doulah et al. 2021; Shahjahan et al. 2021, 2020; Islam et al. 2022a). Elevated temperatures due to climate change can adversely affect native fish species, as temperature fluctuations can significantly affect their overall fitness and growth (D'Abramo and Slater 2019). Furthermore, temperature fluctuations can affect various physiological aspects of fish, including feed ingestion, growth, behavior, reproduction, and energy requirements (Zhao et al. 2020; Maulvault et al. 2018; Ashaf-Ud-Doulah et al. 2019; Naziat et al. 2024). These physiological changes disrupt fish's homeostasis and osmotic and ionic control, leading to increased fish mortality (Shrivastava et al. 2017; Madeira et al. 2016). Additionally, temperature-induced acute and chronic stress can result in metabolic (Fan et al. 2019; Mateus et al. 2017), immunological (Feidantsis et al. 2021; Urbinati et al. 2020), and neuroendocrine impairments in fish (Goikoetxea et al. 2021; O’Donnell 2018).

Hemato-biochemical parameters, such as erythrocytic (nuclear and cellular) abnormalities, and muscle histoarchitecture are valuable indicators of health under heat stress (Islam et al. 2020a; Rebez et al. 2023). Increasing water temperature can significantly disrupt hemato-biochemical parameters and modify erythrocyte and muscle microstructure across different species (Takata et al. 2018; Islam et al. 2020a). Among various hemato-biochemical indices, hemoglobin and blood glucose levels are widely recognized as indicators of stress responses (Shahjahan et al. 2022; Jahan et al. 2019; Ahmed et al. 2016). These indices are significantly influenced by various internal factors such as age, sex, biological behavior, and breeding cycles in species like common carp (*Cyprinus carpio*) and Sobaity seabream (*Sparidentex hasta*) (Golemi et al. 2013; Mozanzadeh et al. 2015). Also, external factors, including food, feeding practices, light, temperature, salinity, and pesticides, also have a significant impact on hemato-biochemical parameters in different species, such as rohu (*Labeo rohita*), Nile tilapia (*Oreochromis niloticus*), and zebrafish (*Danio rerio*) (Jannat et al. 2024; Jahan et al. 2019; Das et al. 2024; Islam et al. 2019b). Among all environmental parameters, temperature is one of the most critical factors for aquatic organisms (Mugwanya et al. 2022). It affects the microstructure of muscle tissue and depletes energy reserves, which, in turn, influence the cell's structure, growth, and muscle position (Steinbacher et al. 2011; Hu et al. 2023). Moreover, cellular and nuclear structural aberrations in erythrocytes can cause DNA damage by releasing DNase enzymes and inactivating enzymes essential for DNA repair (Zafalon-Silva et al. 2017).

Acclimation temperature is a widely studied parameter in thermal tolerance research. It refers to the point where fish can adapt themselves to their surrounding environment either for a short or long period (Ashaf-Ud-Doulah et al. 2020; Lagerspetz 2006). Several studies have shown that organisms adapting to different temperatures exhibit varying capabilities to endure sudden thermal variations (Sandblom et al. 2014; Fu et al. 2018). Moreover, temperature fluctuations directly influence the oxygen consumption rate of organisms, significantly affecting their metabolic activity (Salvato et al. 2001; Islam et al. 2020c, 2022b). For example, Brix et al. (2004) reported that the hemoglobin content of fish blood increased during adaptation to hypoxic conditions in response to temperature elevation.

In nature, fish in open water bodies tend to move from lower to higher latitudes by following species-specific patterns in response to increased water temperature (Alabia et al. 2018; Fogarty et al. 2017). However, farmed fish in closed-water bodies (ponds, lakes, etc.) cannot behaviorally cope with such temperature alterations. Therefore, investigating the effects of temperature fluctuations on fish's critical thermal tolerance and acclimation has become an essential procedure for researchers to explore thermal tolerance (higher and lower) in aquatic organisms, often until equilibrium loss or lethal point.

Nile tilapia is a widely cultured species and, based on world production, ranks second after carp (Rahman et al. 2021a). This tropical species can tolerate a broad range of temperatures (11–42 °C), both in natural and farmed conditions (FAO 2023). In the wild, this species inhabits a variety of temperate regions, from warmer to cooler areas, in lakes, rivers, or other open water bodies. At the same time, in farming conditions, the temperature is typically maintained within an optimal range for maximum growth, between 22 and 29 °C (Mjoun et al. 2010). In the wild, the lower and upper lethal temperature ranges are between 11/12 °C and 42 °C (Kour et al. 2014), which can lead to reduced growth, delayed maturation, or mortality. Some unique features, such as the capability to adapt to a wide range of environmental changes (Singha et al. 2021), the high acceptability of commercial feeds (Ogello et al. 2014), the presence of fewer intermuscular bones (Moesch et al. 2016), the short culture period (Francis and Esa 2016), and the strong immune capacity against diseases (Al-Deriny et al. 2020; Foysal et al. 2020) make this species attractive to farmers and consumers alike.

An increase in water temperature negatively affects Nile tilapia by inducing thermal stress. This stress occurs when environmental temperature exceeds the species' upper or lower thermal limits, placing aquatic animals in critical conditions (Collier et al. 2019; Kazmi et al. 2022). Critical thermal maxima (CTmax) have been used since the 1950s to assess the acute thermal tolerance in fishes (Desforges et al. 2023). These conditions have significant ecological impacts not only on fish but also on various other aquatic species. Such stress results in serious physical consequences, including impaired growth and development, alterations in the immune system, reproductive issues, oxidative stress, and degraded meat quality (Li et al. 2024; Mariana and Badr 2019; Ma et al. 2023). Moreover, they can disrupt ovarian tissues, hormone synthesis, elevate stress hormone levels such as cortisol, induce muscle protein breakdown, and increase reactive oxygen species production (Zhang et al. 2025; Naziat et al. 2024; Tang et al. 2022). Consequently, it is essential to adopt effective strategies to conserve aquaculture species and promote sustainability while maintaining ecological balance amid changing thermal conditions.

Recent studies have focused on the effects of temperature on Nile tilapia under various conditions to evaluate their physiological responses. For example, Islam et al. (2020b) investigated the critical thermal tolerance limits of Nile tilapia in both hypoxic and normoxic conditions. Another study highlighted the impact of chronic temperature exposure (31 °C, 34 °C, and 37 °C) on growth performance, hemato-biochemical parameters, and erythrocyte structures in Nile tilapia across different temperature regimes (Islam et al. 2020a). Despite these efforts, significant knowledge gaps remain regarding the physiological responses of Nile tilapia to acute thermal stress across different acclimation temperatures. Therefore, fish were acclimated to various temperature regimes (31 °C, 34 °C, and 37 °C) before initiating the acute temperature exposure. The use of CTmax has previously been applied to various species, including rohu and zebrafish (Jannat et al. 2024; Ashaf-Ud-Doulah et al. 2020). Consequently, we chose Nile tilapia to assess acute thermal tolerance. The current study aims to improve understanding of stress responses and physiological conditions in Nile tilapia by examining several key hemato-biochemical parameters, erythrocytic (nuclear and cellular) abnormalities, muscle histoarchitecture, as well as CTmax following acute exposure to elevated temperatures.

## Materials and methods

### Ethical statement

The experimental procedures and animal handling were carried out following the guidelines of the Animal Act and Welfare (BAU-FoF/2021/002). The ethical committee of Bangladesh Agricultural University, Mymensingh, Bangladesh, approved the experimental protocol. The study was conducted after obtaining approval from the university's statutory authority.

### Experimental fish

Healthy Nie tilapia (*O. niloticus*) fingerlings, without any visible external lesions, were obtained from Sharnalata Agro Fisheries Limited in Fulbaria, Mymensingh, Bangladesh. The initial length and weight of the fingerlings were 7.2 ± 0.42 cm and 8.4 ± 0.61 g, respectively. All fingerlings were acclimated in 100 L glass aquaria under laboratory conditions. During the two-week acclimation at 31 °C, no thermostat controller was used, and the fish were allowed to maintain naturally occurring temperature conditions. After the two-week acclimation, the fish were distributed into three tanks to adapt them to three different water temperature regimes. The photoperiod was maintained at 12:12 h. They were fed a commercial feed containing 40% protein, 7% lipid, 25% carbohydrate, 12% moisture, 12% ash, and 4% crude fiber (Mega Feed Ltd., Dhaka) (Islam et al. 2020b). The fish were fed twice daily to satiation, and regular siphoning was performed to maintain water quality by removing uneaten feed particles and feces.

### Determination of the high-temperature tolerance limit of Nile tilapia

A total of 120 fingerlings were randomly distributed into glass aquariums measuring 75 cm × 45 cm × 45 cm and equally distributed to different temperature treatments: 31 °C, 34 °C, and 37 °C. Each aquarium contained 100 L of clean tap water, maintaining a stocking density of 40 fingerlings per treatment group. The target temperatures of 34 °C and 37 °C were reached by gradually increasing the water temperature by 1 °C each day, starting from the initial control temperature of 31 °C. Then, all fingerlings were again acclimated to three temperature regimes at 31 °C, 34 °C, and 37 °C for a week. During this acclimation period, a filter (Sebo-aquarium Internal Filter WP-850F from Yiwu Nihao Aquarium, Zhejiang, China) was used to maintain clean water in the glass aquarium. During this period, no water was changed, as the filter was sufficient to keep the water clean.

**Fig. 1:**
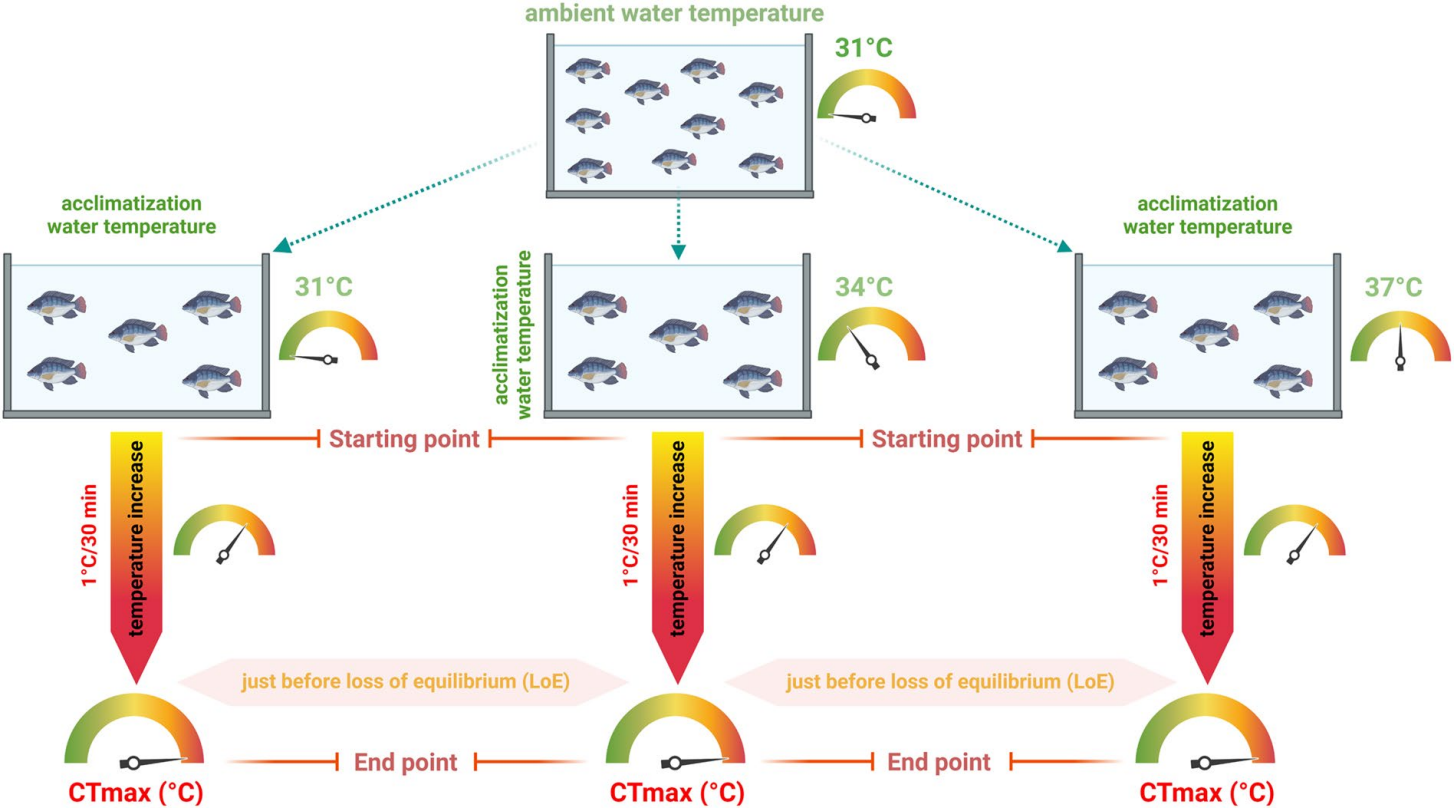
Schematic diagram of the experimental design to determine the high-temperature tolerance limit (CTmax) of fish acclimated to three temperature conditions. A thermostat was used to carefully monitor and increase the temperature throughout the process. Blood samples were collected from the sampled fish at both the starting and ending points of each condition

The assessment of critical thermal tolerance in the experiment was conducted using a methodology outlined in previously published literature (Ashaf-Ud-Doulah et al. 2020). Specifically, a thermostat controller (Model: REI-SEA, 300 watts, Japan) was employed to gradually increase the temperature and maintain it at the target degree. To ensure accuracy and consistency, the water temperature was regularly monitored with a digital Celsius thermometer (Model: HI-98107, Hanna, Romania). Subsequently, ten fingerlings were randomly selected from each temperature group (31 °C, 34 °C, and 37 °C) and distributed equally into separate aquariums to determine Nile tilapia's critical thermal maxima (CTmax). Three replicates were used to determine the CTmax of each temperature group, following a similar procedure in all replicates. To determine the CTmax, all aquaria holding 50 L of water with dimensions 50 cm × 35 cm × 35 cm were used, and the water temperature in each was gradually increased by 1 °C every 30 min. During the experimental period, approximately 80% oxygen saturation was maintained using an air pump to prevent hypoxia. The CTmax was considered reached when the experimental fish exhibited signs of distress, such as unusual movement, floating upside down, scratching against the aquarium wall, or positioning themselves upside down in the water column. This point was recorded as the endpoint (Fig. 1).

### Sampling points

During the high-temperature tolerance stage, hematological and biochemical parameters were analyzed from sampled fish. To do this, five fish samples were collected at the beginning from each tank, just before the temperature increase, and the remaining five fish at the end, when the fish were about to die due to losing equilibrium. Then 5 mg/L clove oil was used for anesthesia (Hilltech Canada Inc. Vankleak Hill. Ontario, Canada) to collect blood samples only at the starting point. To evaluate the muscle fiber architecture, dorsal muscle tissues were collected from each fish only at the end of the sampling point.

### Measurement of hemato-biochemical parameters

Blood samples were collected from the caudal vein using a heparinized plastic syringe to measure the hemato-biochemical indices. After collection, the samples were preserved by storing them in microfuge tubes containing an anticoagulant (20 mM EDTA solution, Reagent Mart, Dhaka, Bangladesh). Hemoglobin (Hb) levels (g/dL) and blood glucose levels (mg/dL) were measured using hemoglobin and glucose strips, respectively, in a digital EasyMate® GHb (Model: ET-232, Bioptik Technology Inc. Taiwan 3507). A Neubauer hemocytometer (Blaxhall and Daisley) connected to a light microscope was used to quantify red blood cells (RBCs) and white blood cells (WBCs).

### Cellular and nuclear abnormalities of peripheral erythrocytes

The analysis of erythrocytic nuclear abnormalities (ENAs) and erythrocytic cellular abnormalities (ECAs) was conducted following the methodology outlined in previous studies (Jahan et al. 2019; Shahjahan et al. 2019; Islam et al. 2026). Blood samples were initially collected at the beginning and end stages of the thermal tolerance test. Immediately after collection, blood smears were prepared on sterile slides and dried at room temperature for 10 min. These prepared smears were then fixed using methanol (Merck KGaA, Damstadt, Germany) for 10 min. The fixed smears were subsequently stained with 5% Giemsa stain solution (D Lab Chemicals Dhaka; License No. 782), rinsed with distilled water, air dried overnight, and finally mounted using the mounting agent DPX (Fisher Scientific UK, Bishop Meadow Road, Loughborough). ENAs and ECAs were observed using an electronic microscope (MCX100, Micros Austria). Three blood smear slides were prepared for each fish sample, and 2000 cells were counted from each slide.

### Histomorphology assessment of muscle

Dorsal muscle tissues were collected from each treatment group to assess the muscle architecture and processed for histomorphology evaluations. The collected tissue samples were immediately fixed at 10% buffered formalin and then dehydrated at different graded alcohol dilutions. The tissue samples were treated with xylene and embedded in paraffin wax. Embedded tissue blocks were sectioned manually with a rotary microtome (Thermo Scientific, Shandon, Finesse ME; SI: FN1021M9812; New Hampshire, United States) at 5 µm thickness. Then, the sectioned slides were stained with hematoxylin and eosin (H & E) following the standard protocols. The stained slides were then monitored under a digital light microscope (Micros 100X, Austria, AmScope 1000).

### Data analysis and statistics

R software (version 4.2.2) and R Studio were used for statistical analysis on Windows 10 (version 22H2; OS Build 19,045.3208). A two-way ANOVA was performed to assess the significant variations (p < 0.05) in hematobiochemical parameters and erythrocyte frequencies among different temperature treatments. Finally, Tukey’s post hoc multiple comparison test was employed to evaluate significant variations in muscle histomorphometry and tolerance limits among the experimental groups. The Kruskal–Wallis test was used for non-parametric data analysis. All values are presented as mean ± standard deviation (SD).

### Principal component analysis

Principal Component Analysis (PCA) was performed using the 'R' software package "ggfortify". All hematobiochemical parameters at the endpoint were used to assess the effects of acute temperature.

### Pearson’s correlation analysis

Pearson’s correlation was employed to correlate CTmax and acclimated temperature using the ‘R’ software followed by the “ggplot2” package.

## Results

### Changes in hemato-biochemical parameters

Table 1 shows the hemato-biochemical status of fish at the upper thermal tolerance stage. The results indicate a significant decrease in blood Hb levels and red blood cell (RBC) content at all acclimation temperatures at the endpoint of thermal tolerance. These parameters were significantly reduced (p < 0.05) at the highest temperature (37 °C) compared to the lowest temperature (31 °C) at both the initial and final points of thermal tolerance. On the other hand, white blood cell (WBC) and blood glucose levels showed opposite trends at the endpoint of thermal tolerance across all acclimated temperatures. There was a significant (p < 0.05) increase in blood glucose levels at the highest temperature (37 °C) compared to the lowest temperature (31 °C) at both the beginning and end points of thermal tolerance.

**Table 1:** Changes in hemato-biochemical parameters during the determination of high-temperature tolerance of Nile tilapia

Parameters	Sampling point	Acclimation temperature (°C)			
		31	34	37	p-value
Hb (g/dL)	Start	9.55 ± 0.31^b,2^	8.50 ± 0.31^b,12^	8.11 ± .19^b,1^	2.79e-05
	End	7.78 ± 0.97^a,2^	7.06 ± 0.52^a, 2^	6.44 ± 1.06^a,1^	3.55e-06
RBC (× 10^6^/mm^3^)	Start	1.84 ± 0.16^b,2^	1.44 ± 0.14^b,12^	1.33 ± .16^b,1^	0.00285
	End	1.28 ± 0.39^a,2^	1.14 ± 0.47^a,12^	1.03 ± 0.33^a,1^	0.033
WBC (× 10^3^/mm^3^)	Start	1.45 ± 0.10^a,1^	1.64 ± 0.14^a,1^	1.84 ± 0.14^a,1^	0.0645
	End	1.68 ± 0.12^b,1^	1.95 ± 0.17^b,1^	2.23 ± 0.16^b,1^	0.108
Blood glucose (mg/dL)	Start	106.82 ± 16.97^a,1^	121.25 ± 15.73^a,12^	149.50 ± 11.87^a,2^	0.00322
	End	155.66 ± 16.06^b,1^	215.8 0 ± 27.67^b,12^	245.4 ± 68.09^b,2^	0.0368

Values of a single hemato-biochemical parameter (at the start and end) in a column with different alphabetical superscripts are significantly different (p < 0.05). Values with different numeric superscripts in a row differ significantly (p < 0.05) among acclimation temperatures (ºC). All values are expressed as mean ± SD (n = 15)

### Changes in the cellular and nuclear morphology of blood cells

**Fig. 2:**
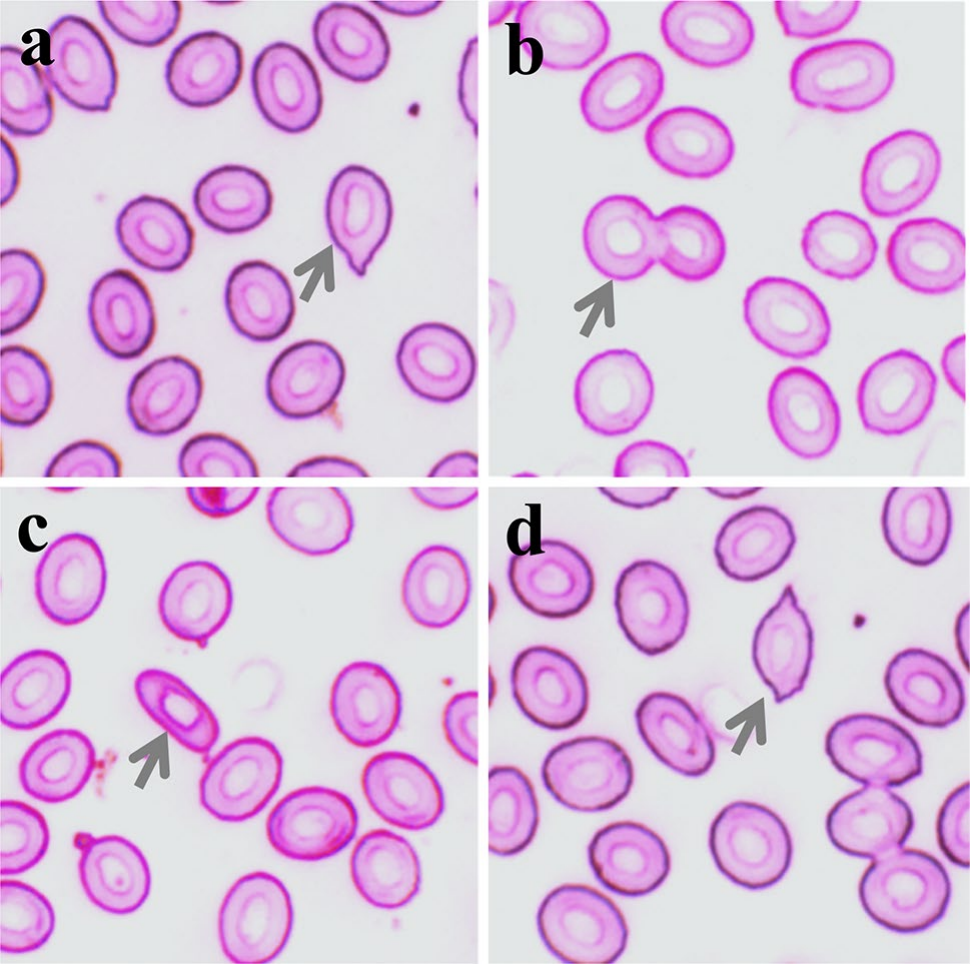
Erythrocytic cellular abnormalities (ECAs) in Giemsa-stained blood smears of Nile tilapia were observed during the determination of high-temperature tolerance limits while acclimated at three different temperatures: **a** tear-drop, **b** twin, **c** elongated, and **d** spindle-shaped

In our study, we noticed various abnormalities in the red blood cells of fish when exposed to high temperatures. The ECAs included unusual shapes such as teardrop, twin, elongated, and spindle (Fig. 2). The frequency of all types of these abnormalities was significantly higher at the end point of thermal tolerance across all acclimated temperatures than at the starting point (Table 2).

**Table 2:** Cellular abnormalities of erythrocytes during the determination of high-temperature tolerance of Nile tilapia

Abnormalities	Sampling point	Acclimation temperature (°C)		
		31	34	37
Tear-drop	Start	0.21 ± 0.09^a,1^	0.36 ± 0.17^a,12^	0.79 ± 0.71^a,2^
	End	0.79 ± 0.41^b,1^	1.13 ± 0.11^b,12^	1.30 ± 0.82^b,2^
Twin	Start	0.47 ± 1.09^a,1^	3.46 ± 0.25^a,2^	3.55 ± 0.97^a,2^
	End	2.67 ± 0.23^b,1^	5.66 ± 0.73^b,2^	6.78 ± 0.32^b,2^
Elongated	Start	0.53 ± 0.16^a,1^	1.82 ± 0.09^a,2^	2.18 ± 0.22^a,2^
	End	2.19 ± 0.10^b,1^	3.15 ± 0.33^b,2^	3.26 ± 0.19^b,2^
Spindle	Start	0.47 ± 1.04^a,1^	1.41 ± 0.21^a,2^	1.52 ± 0.37^a,2^
	End	1.67 ± 0.21^b,1^	2.16 ± 0.72^b,12^	3.78 ± 0.22^b,2^

Values of a single abnormality (at the start and end) in a column with different alphabetical superscripts are significantly (p < 0.05) different. Values with different numeric superscripts in a row differ significantly (p < 0.05) among acclimation temperatures (ºC). All values are expressed as mean ± SD (n = 15)

**Fig. 3:**
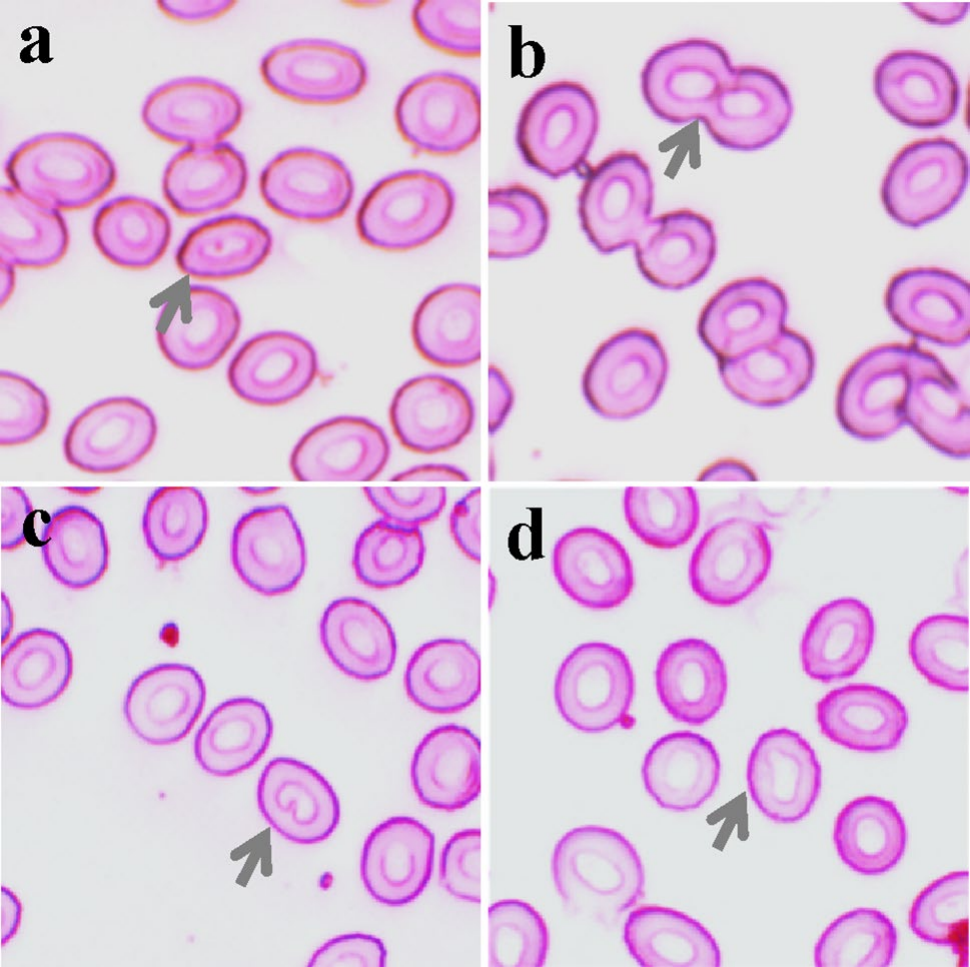
Erythrocytic nuclear abnormalities (ENAs) were observed in Giemsa-stained blood smears of Nile tilapia during the determination of high-temperature tolerance limit while acclimated at three temperatures: **a** karyopyknosis, **b** nuclear bridge, **c** notched, and **d** nuclear bud

Additionally, we observed various ENAs such as karyopyknosis, nuclear bridge, notched, and nuclear bud during exposure to high temperatures (Fig. 3). The frequencies of these cellular and nuclear abnormalities were significantly higher at the final point of thermal tolerance in all acclimated temperatures compared to the starting point (Table 3). Furthermore, within the treatments, the frequencies of these abnormalities were significantly higher at the highest acclimation temperature (37 °C) compared to the lowest acclimation temperature (31 °C) at both the beginning and end points of the study (Table 3).

**Table 3:** Nuclear abnormalities of erythrocytes during the determination of high-temperature tolerance of Nile tilapia

Abnormalities	Sampling point	Acclimation temperature (°C)		
		31	34	37
Karyopyknosis	Start	0.29 ± 0.07^a,1^	0.59 ± 0.17^a,12^	1.46 ± 0.28^a,2^
	End	1.73 ± 0.11^b,1^	2.26 ± 0.05^b,2^	3.73 ± 1.01^b,2^
Nuclear bridge	Start	0.12 ± 0.06^a,1^	0.29 ± 0.10^a,12^	0.63 ± 0.13^a,2^
	End	0.59 ± 0.09^b,1^	0.73 ± 0.11^b,12^	0.94 ± 0.17^b,2^
Notched	Start	0.21 ± 0.09^a,1^	1.78 ± 0.14^a,2^	2.55 ± 0.10^a,2^
	End	2.11 ± 0.16^b,1^	3.22 ± 0.25^b,2^	4.63 ± 0.31^b,2^
Nuclear bud	Start	0.16 ± 0.03^a,1^	0.28 ± 0.10^a,12^	0.92 ± 0.13^a,2^
	End	0.37 ± 0.07^b,1^	0.63 ± 0.11^b,12^	0.97 ± 0.17^b,2^

Values of a single abnormality (at the start and end) in a column with different alphabetical superscripts are significantly (p < 0.05) different. Values with different numeric superscripts in a row differ significantly (p < 0.05) among acclimation temperatures (ºC). All values are expressed as mean ± SD (n = 15)

### Histomorphology of muscle

**Fig. 4:**
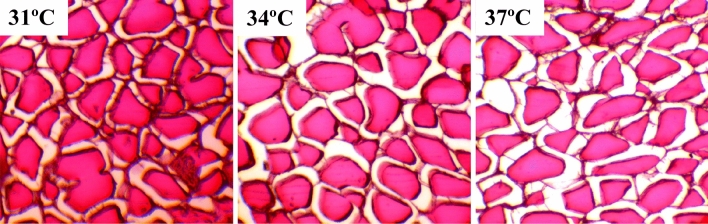
Effects of temperature on the histoarchitecture of muscle fiber in Nile tilapia at the end point at different temperature regimes

The study demonstrated various histomorphological changes in muscle fibres, with their structure altered as temperature increased (Fig. 4). In the 31 °C group, muscle fibres were mostly healthy and uniform in shape. The bundle of muscle fibres remained intact, and the myoid cell structure was singular and dense. Conversely, in the 37 °C group, the muscle fibres were predominantly elongated and irregular. The muscle fibres experienced fractures, and the space between myocytes increased after heat stress. In the 34 °C group, the muscle fibres appeared to have a more regular, polygonal shape than in the 31 °C group.

### High-temperature tolerance limit

**Fig. 5:**
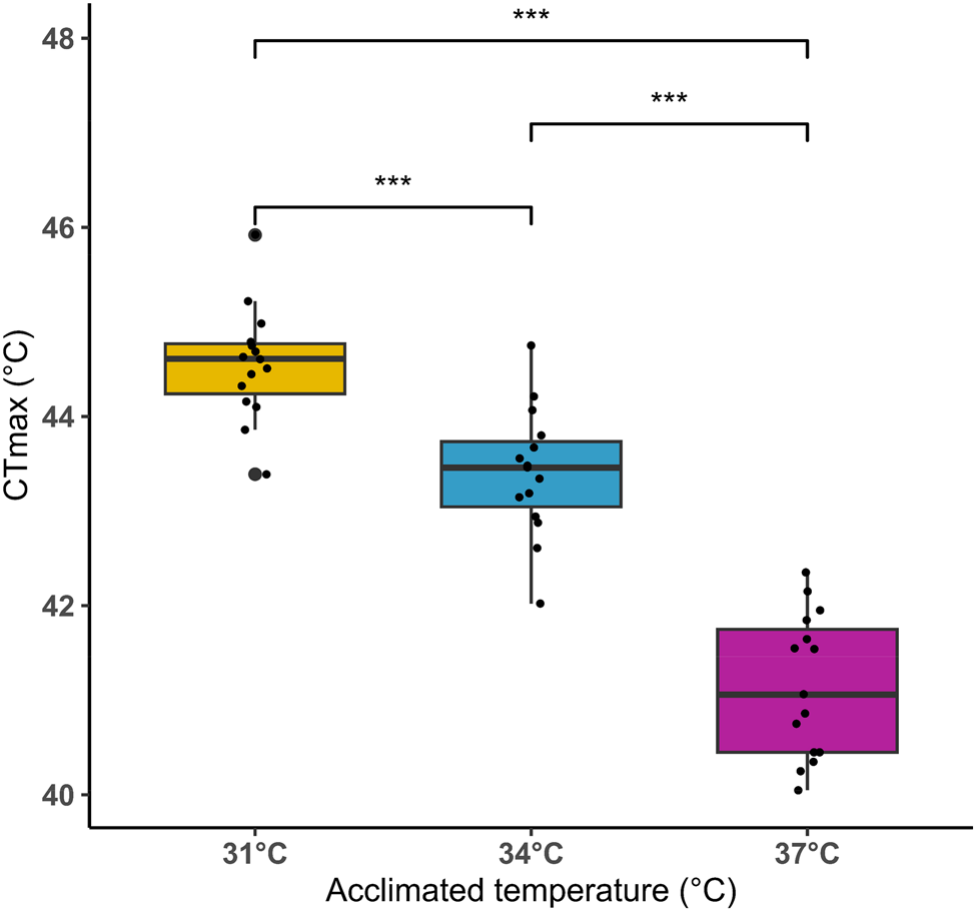
High-temperature tolerance (critical thermal maximum, CTmax) of Nile tilapia acclimated to three temperature conditions. The statistical significance levels among the treatments are denoted by ‘***’, p < 0.001

In this study, the upper thermal limit (CTmax) of *O. niloticus* fingerlings was determined based on the temperature at which the fish lost equilibrium in the aquarium (Fig. 5). The CTmax differed significantly (p < 0.05) among the groups based on the acclimation temperature (31 °C, 34 °C, and 37 °C). The lowest CTmax value of 41.15 ± 0.31 °C was recorded at the highest acclimation temperature (37 °C), compared to the other temperature groups (CTmax value of 43.40 ± 0.17 °C and 44.57 ± 0.21 °C for 34 °C and 31 °C, respectively). For the groups acclimated to different temperatures, the following times were observed for the loss of equilibrium: the group acclimated to 31 °C took 6 h and 47 min, the group acclimated to 34 °C took 4 h and 42 min, and the group acclimated to 37 °C took 2 h and 4.5 min.

### Principal Component Analysis (PCA)

#### Before CTmax

**Fig. 6:**
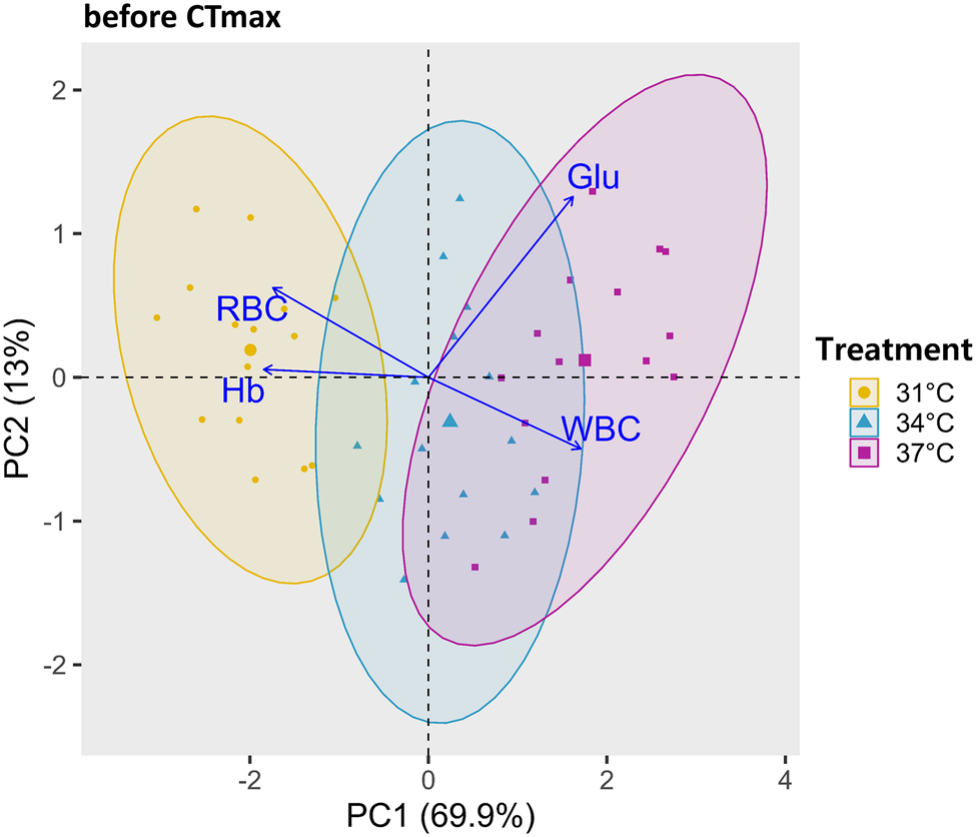
PCA biplot showing the hematobiochemical parameters (RBC; red blood cell, WBC; white blood cell, Hb; hemoglobin, Glu; glucose) of Nile tilapia at the starting point, during the determination of high-temperature tolerance limit when acclimated at three temperatures

Before the CTmax, various hematobiochemical parameters, such as RBC, WBC, Glu, and Hb, are illustrated in the PCA biplot in Fig. 6. In this biplot, the two components, PC1 and PC2, explained 82.9% of the variability in the total dataset. While PC1 accounts for 69.9% and PC2 for 13%, along the PC1 axis, WBC and Glu loadings displayed a positive correlation and contributed most to the variability in the data. Other variables, RBC and Hb, are negatively correlated with PC1 and primarily associated with the PC2 axis. The PCA biplot showed three distinct clusters, reflecting differences among the treatment groups. The 34 °C and 37 °C group clusters appeared similar in size, while the 31 °C group showed the smallest cluster. The 34 °C cluster was positioned near the centre of the PC1 axis, while the 31 °C cluster was located on the negative side of the PC1 axis.

#### After CTmax

**Fig. 7:**
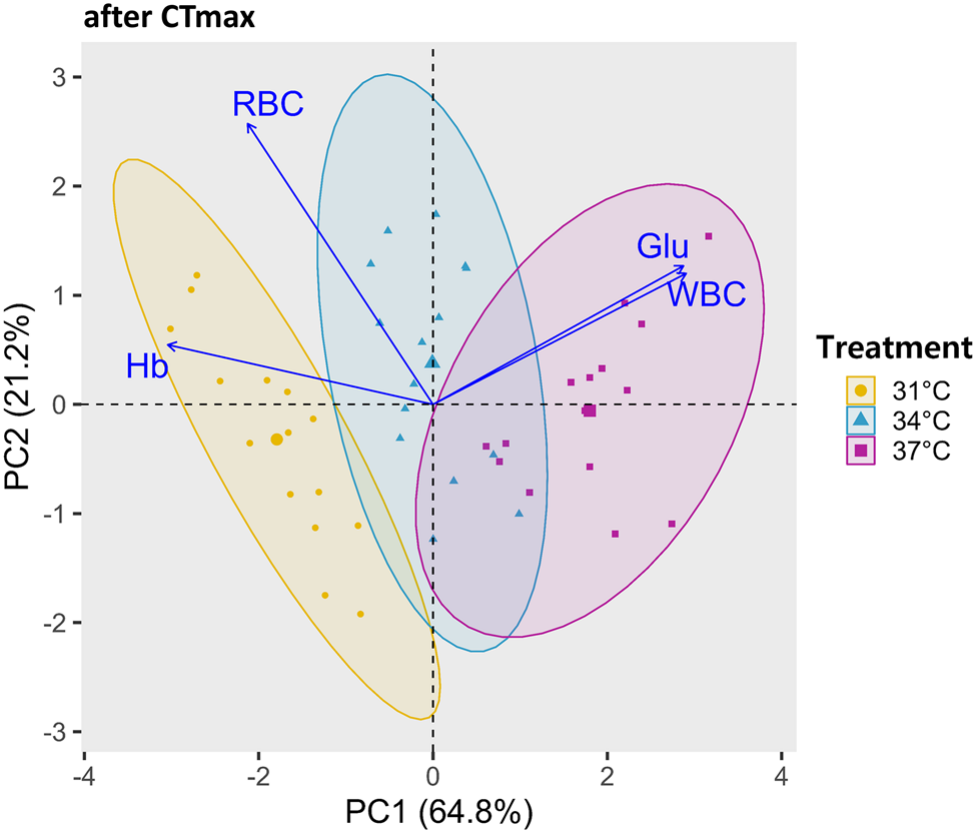
PCA biplot showing the hematobiochemical parameters (RBC; red blood cell, WBC; white blood cell, Hb; hemoglobin, Glu; glucose) of Nile tilapia at the endpoint during the determination of high-temperature tolerance limit when acclimated at three temperatures

Hematobiochemical parameters at the end of the study, including RBC, WBC, Glu, and Hb, are shown in Fig. 7. Among these variables, Glu and WBC exhibit a positive correlation with PC1. In contrast, RBC and Hb are negatively correlated. The WBC variable has the highest loading on PC1. PC1 explains 64.8% of the data's variability, while PC2 accounts for 21.2%. The PCA biplot displays three distinct clusters, with the 37 °C cluster being the largest compared to the others, while the 34 °C cluster appears to be intermediate along the PC1 axis. The 31 °C cluster is somewhat distinct from the others and lies on the negative side of the PC1 axis.

### Pearson’s correlation

**Fig. 8:**
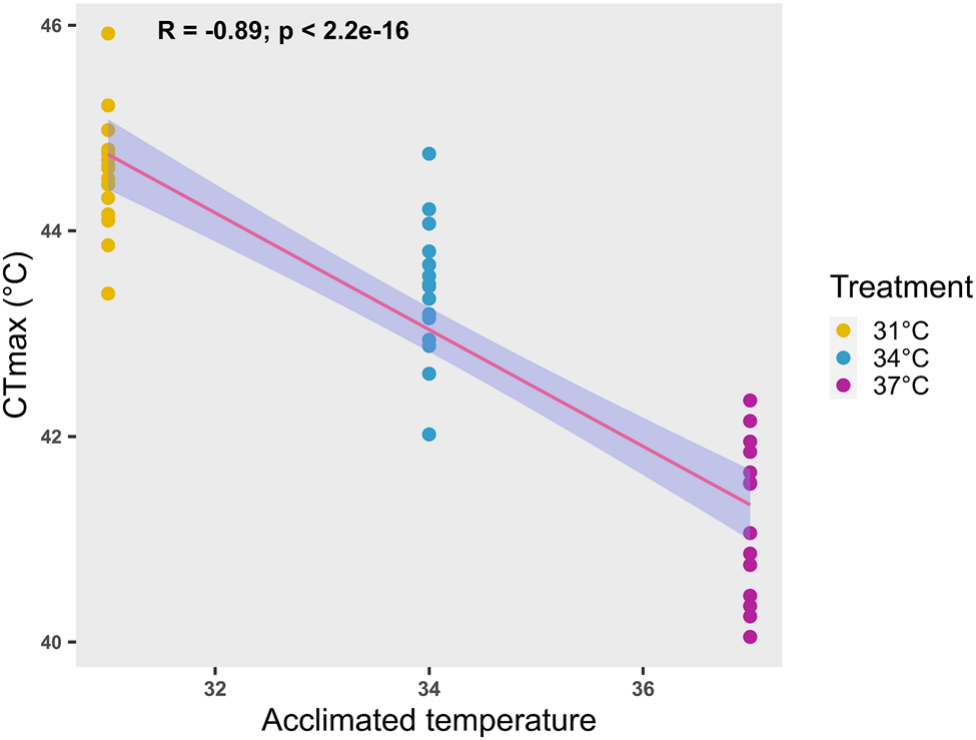
Pearson’s correlation analysis, conducted to examine the relationship between critical thermal maxima (CTmax) and acclimated temperature (°C), highlights the acclimation response. Individual CTmax values for each fish are represented by large dots and categorised according to their respective acclimated temperature groups. The analysis reveals a negative correlation between CTmax and acclimated temperature, indicating that Nile tilapia's thermal tolerance limit decreases as acclimated temperature increases

Pearson's correlation analysis was performed to evaluate the relationship between CTmax and different acclimation temperatures (Fig. 8). The results revealed that CTmax was highest at the lowest acclimation temperature (31 °C), but decreased significantly (r = -0.89; p = 2.2e-16) as the acclimation temperature increased.

## Discussion

Each fish species has a specific range of water quality parameters in which it thrives and performs optimally. Any deviation from this range, whether an increase or a decrease, can cause stress and significantly affect the fish's physiology. Among these parameters, temperature is crucial at all stages of a fish's life cycle, as thermal stress can disrupt their normal functions (D'Abramo and Slater 2019).

In our study, blood samples were collected before and after CTmax to gain insight into the physiological response to thermal stress. This approach has been previously used in various species, including zebrafish, rohu, and Nile tilapia (Jannat et al. 2024; Ashaf-Ud-Doulah et al. 2020; Islam et al. 2020b), and can serve as biomarkers for aquaculture or ecological monitoring, especially for assessing thermal stress. Blood is a key indicator of stress and can be directly affected by factors such as temperature, acclimation period, or other environmental conditions (Ribeiro et al. 2018). Measuring changes in parameters such as Hb, RBC, Glu, and WBC offers valuable insights into an organism’s physiological response to heat and overall health during thermal tolerance (Dagoudo et al. 2023; Islam et al. 2020a). Analysing these parameters at different temperature points provides important information on the upper thermal limits for Nile tilapia, which could assist the industry or fish farms in developing more temperature-tolerant fish.

A significant decrease in Hb and RBC levels was observed as acclimation temperature increased. Similar patterns were also detected at the endpoint when compared to the starting point. This is likely because higher water temperatures reduce the affinity and solubility of oxygen in aquatic environments, potentially leading to oxygen depletion for fish. Moreover, elevated temperatures can increase ammonia toxicity levels. To cope with these adverse conditions, fish may lower their Hb and RBC levels to mitigate oxidative damage caused by higher temperatures (Varghese et al. 2021; Xu et al. 2021). One plausible explanation for the reduction in these parameters may be linked to damage to the hematopoietic system under stress conditions (Alak et al. 2023). Similar findings have been reported in other species, including *Pangasianodon hypophthalmus* (Phuc et al. 2017), *Labeo rohita* (Ashaf-Ud-Doulah et al. 2019), and *Takifugu obscurus* (Cheng et al. 2017).

Current research indicates that the WBC count in Nile tilapia notably increases at higher acclimation temperatures (37 °C). This rise is probably related to a stress response caused by exposure to elevated temperatures. The increased number of WBCs may reflect heightened antibody production, aiding the fish in managing stressful environmental conditions (Begg and Pankhurst 2004; Shahjahan et al. 2018). Other studies also suggest that a higher WBC count is linked to fish defence mechanisms and immune responses. Conversely, elevated temperatures have been reported to decrease white blood cell counts in *Gasterosteus aculeatus* (Dittmar et al. 2014), *Epinephelus coioides* (Chen et al. 2019), and *Tor putitora* (Akhtar et al. 2013a). These discrepancies could be due to differences in stress coping capabilities among species, as well as variations in exposure durations.

An increase in blood glucose levels in fish is a key indicator of stressful conditions (Hossain et al. 2019; Eslamloo et al. 2012; Jentoft et al. 2005). Under heat stress, the fish's endocrine system activates the hypothalamic-pituitary-interrenal axis, which is responsible for producing and releasing stress hormones such as cortisol and catecholamines (Wendelaar Bonga 1997). This response also stimulates an increase in lactate and glucose mobilisation for energy, preparing the fish for immediate action and triggering physiological responses (Balta et al. 2017; Bard and Kieffer 2019). Glucose is produced through gluconeogenesis (Nakano et al. 2014), resulting in higher blood glucose levels (Jentoft et al. 2005; Bögner et al. 2018; Viegas et al. 2011). Elevated glucose levels in fish blood support metabolic processes, as glucose is a primary energy source, which is vital for maintaining homeostasis under stressful conditions (Jiang et al. 2017; Lupatsch et al. 2010; Nakano et al. 2014). Recent research has shown that blood glucose levels in zebrafish increased when exposed to higher temperatures (Jannat et al. 2024). This rise likely reflects an increased rate of gluconeogenesis to meet the higher energy demands during thermal stress (Alexander et al. 2011; Akhtar et al. 2013b). As ectothermic animals, fish's metabolic rates and physiological functions are directly affected by rising temperatures (Volkoff and Rønnestad 2020). To cope with this challenging situation, fish may seek out cooler microhabitats to help regulate their physiology and maintain normal functions, ultimately leading to increased glucose levels to provide additional energy (Malini et al. 2018; Islam et al. 2019a). Furthermore, high temperatures can suppress the fish's respiratory metabolism, causing them to rely on intracellular glycogen. As a result, the fish release hyperglycemic hormones that break down glycogen and elevate glucose levels, leading to hyperglycemia (Banaee et al. 2011).

Exposure to high temperatures not only alters the number of blood cells but also causes significant changes in their shape and structure (Jannat et al. 2024). In this study, various erythrocyte abnormalities, including teardrop, twin, elongated, and spindle shapes, as well as nuclear abnormalities such as karyopyknosis, nuclear bridges, notches, and nuclear buds, were identified. These abnormalities were observed more frequently in the group exposed to the highest acclimated temperatures. Numerous studies have demonstrated similar findings in groups subjected to elevated temperatures (Rahman and Baek 2019; Panase et al. 2018; Islam et al. 2020c).

The presence of abnormal erythrocytes may result from the unequal distribution of hemoglobin (Mekkawy et al. 2011). High temperatures can increase the concentration of enzymes associated with lipid peroxide products, which may lead to blood cell abnormalities (Ghaffar et al. 2015). Oxidative stress induces changes in the morphology of blood cells in various ways, affecting the synthesis of cytoplasmic content and the cell membrane (Ghaffar et al. 2015; Obeagu et al. 2024). Additionally, high temperatures disrupt lipid solubility in the erythrocyte membrane, potentially leading to apoptosis (Walia et al. 2013).

Collagen and the morphological features of muscle fibres are important indicators of fish flesh texture (Wei et al. 2016; Aussanasuwannakul et al. 2012). By the end of the trial, the muscle fibres in fish acclimated at 31 °C appeared regular in shape. In contrast, those in the group acclimated to 37 °C showed signs of partial denaturation, reduced size, and irregular shape. This suggests that collagen content in the muscle may decrease as temperature increases. The reduction in muscle fibres could partly explain the observed decline in growth and changes in other biochemical parameters at 37 °C, as shown in earlier studies (Jannat et al. 2024; Scott and Johnston 2012). These findings indicate that high temperatures can notably affect the structure of fish muscle and collagen deposition, potentially altering muscle quality. This implies that the regulatory mechanisms controlling collagen in muscle fibres at different temperatures may be linked to various physiological functions.

Critical thermal maxima (CTmax) is an ecological index used to predict the adverse effects of thermal stress on species. It serves as an important physiological reference and an early indicator of thermal stress (Stewart and Allen 2014). In the current study, Nile tilapia exhibited higher CTmax values at lower acclimatized temperatures (31 °C and 34 °C) than at 37 °C. Higher temperatures were associated with lower CTmax values, suggesting a negative relationship between temperature and CTmax. Similar findings were reported by Ashaf-Ud-Doulah et al. (2020), who noted that warmer temperatures decreased CTmax values in rohu. Conversely, an opposite trend was observed in Nile perch, where fish acclimated to higher temperatures exhibited higher CTmax values (Nyboer and Chapman 2017).

The proposed physiological mechanisms indicate that rapid thermal stress in fish is affected by three primary factors: reaction rates, protein structure, and membrane fluidity (Ern et al. 2023). During acute thermal stress, changes in these molecular structures can cause a loss of homeostasis and ultimately lead to death through various cellular, organ, and physiological pathways. These pathways may include mitochondrial dysfunction, oxygen limitation, and altered responses of excitable cells, which can result in neural or muscular failure. Evidence of oxygen limitation has been observed in some species, while others may not show this under certain conditions (Ern et al. 2023). Additionally, changes in CTmax values are affected by factors such as fish age, species, thermal rate changes, environmental thermal characteristics, and the fish's condition factor (Ern et al. 2023; Rahman et al. 2021b; Morita et al. 2010). Multiple studies have demonstrated that even slight variations in fish body size can significantly influence the thermal preferences of species (Christensen et al. 2020; Elliott and Allonby 2013). For example, in the present study, 8.4 g Nile tilapia had a maximum CTmax of 44.57 °C. Conversely, the same species with a larger body weight of 85.4 g showed a maximum CTmax of 38.8 °C, which is lower than our current finding (Leonard and Skov 2022). Other critical factors, such as acclimation temperature and the rate of thermal ramping, can also impact thermal limits (Desforges et al. 2023). Acclimation temperature influences CTmax and is commonly utilised to evaluate the thermal sensitivity of ectothermic animals (Stewart et al. 2023).

Numerous studies have explored how acclimation affects CTmax. According to the existing literature, higher acclimation temperatures tend to increase CTmax in various fish species, including juvenile grass carp, red-spotted grouper, and brook trout (Khan et al. 2024; Stewart et al. 2023; Rahman et al. 2021b); however, our study shows a different result. This might be due to the short-term nature of the acclimation temperature. Another possible reason could be the rapid temperature changes during the study. Elevated temperatures cause stress responses in Nile tilapia during and after exposure to 37 °C (Islam et al. 2020a). In our experiment, the temperature increased by 1 °C every 30 min, which may have caused acute stress in the high-acclimation-temperature group. Therefore, our findings indicate that short-term acclimation to higher temperatures may reduce Nile tilapia's thermal tolerance.

In the PCA biplot (after CTmax), PC1 accounted for most of the variance in the hematobiochemical dataset, indicating that white blood cell (WBC) and glucose (Glu) levels increased sharply as temperature rose, exposing the fish to critical temperatures. During this acute heat challenge, the fish's survivability is limited, and they experience acute stress. The increase in WBC and Glu levels indicates a response to stress conditions.

The slope in Pearson’s correlation shows a negative association between CTmax and temperature, meaning that the value of CTmax decreased as the temperature increased. This negative relationship may be due to the direct negative effect of temperature on hematobiochemical, physical, or metabolic reactions. This study confirms that environmental factors, such as temperature, induce acute stress and affect fish physiology and health status.

## Conclusion

The results of the present study confirm that acute thermal stress during short-term acclimation influences the biochemical parameters, erythrocytes and muscle structures, and the upper thermal limits of Nile tilapia. The CTmax depends on acclimation manipulation, and more molecular studies underlying the mechanisms driving acute thermal stress are recommended. Assessing the thermal tolerance limit has become increasingly important in ecology to predict the stress levels of various physiological conditions in aquaculture species. These results can be valuable for species selection, optimising culture, and improving management practices for farmed fish, especially in light of climate change. Proper monitoring, modern technology, and producing high-temperature tolerant strains through selective breeding could be recommended to mitigate acute thermal stress. Moreover, enhancing immunity and mucosal health through the use of probiotics and other components (such as microalgae and insect meal) in aquafeeds may help reduce acute thermal stress for aquatic farmed species.

## Data Availability

The data that support the findings of this study are available on request from the corresponding author [Ioannis N. Vatsos].
